# Increased Precursor Cell Proliferation after Deep Brain Stimulation for Parkinson's Disease: A Human Study

**DOI:** 10.1371/journal.pone.0088770

**Published:** 2014-03-03

**Authors:** Vinata Vedam-Mai, Bronwen Gardner, Michael S. Okun, Florian A. Siebzehnrubl, Monica Kam, Palingu Aponso, Dennis A. Steindler, Anthony T. Yachnis, Dan Neal, Brittany U. Oliver, Sean J. Rath, Richard L. M. Faull, Brent A. Reynolds, Maurice A. Curtis

**Affiliations:** 1 Department of Neurosurgery, McKnight Brain Institute, University of Florida, Gainesville, Florida, United States of America; 2 Department of Neurology, UF Center for Movement Disorders and Restoration, University of Florida, Gainesville, Florida, United States of America; 3 Department of Anatomy with Radiology and Centre for Brain Research, School of Medical and Health Sciences, The University of Auckland, Auckland, New Zealand; 4 Department of Pathology, University of Florida, Gainesville, Florida, United States of America; 5 Department of Biostatistics, University of Florida, Gainesville, Florida, United States of America; Emory University, Georgia Institute of Technology, United States of America

## Abstract

**Objective:**

Deep brain stimulation (DBS) has been used for more than a decade to treat Parkinson's disease (PD); however, its mechanism of action remains unknown. Given the close proximity of the electrode trajectory to areas of the brain known as the “germinal niches,” we sought to explore the possibility that DBS influences neural stem cell proliferation locally, as well as more distantly.

**Methods:**

We studied the brains of a total of 12 idiopathic Parkinson's disease patients that were treated with DBS (the electrode placement occurred 0.5–6 years before death), and who subsequently died of unrelated illnesses. These were compared to the brains of 10 control individuals without CNS disease, and those of 5 PD patients with no DBS.

**Results:**

Immunohistochemical analyses of the subventricular zone (SVZ) of the lateral ventricles, the third ventricle lining, and the tissue surrounding the DBS lead revealed significantly greater numbers of proliferating cells expressing markers of the cell cycle, plasticity, and neural precursor cells in PD-DBS tissue compared with both normal brain tissue and tissue from PD patients not treated with DBS. The level of cell proliferation in the SVZ in PD-DBS brains was 2–6 fold greater than that in normal and untreated PD brains.

**Conclusions:**

Our data suggest that DBS is capable of increasing cellular plasticity in the brain, and we hypothesize that it may have more widespread effects beyond the electrode location. It is unclear whether these effects of DBS have any symptomatic or other beneficial influences on PD.

## Introduction

Parkinson's disease (PD) is a neurodegenerative disorder causing debilitating tremor, rigidity, bradykinesia and gait disorders and can affect non-motor circuits, causing depression, anxiety and sexual dysfunction [1], [2]. Current treatment options for PD including dopamine replacement (levodopa and dopamine agonists) and surgery (thalamotomy, pallidotomy) only provide symptomatic relief and are not curative. Deep brain stimulation (DBS) is a surgical procedure where leads are stereotactically implanted to reach a specific neural target (subthalamic nucleus (STN), globus pallidus internus (GPi)) and are programmed to deliver chronic electrical stimulation [3], [4], [5]. Close to the lead placement are the germinal zones, the subventricular zone [SVZ] and subgranular zone [SGZ], containing neural stem cells (NSCs) [6], [7], [8]. NSCs can generate new neurons throughout life, which occurs in neurologically normal brains [7], and is increased after neurodegeneration (Huntington's disease, stroke etc) [6], [9], [10], [11], [12], [13], [14], [15], [16], [17], [18]. In PD, a reduction in SVZ dopaminergic innervation results in decreased precursor proliferation [19], although this has been recently challenged [20]. Since the trajectories necessary to reach the target nuclei traverse close to the lateral and third ventricles [21] we investigated the effects of DBS on neural precursor cell proliferation in the SVZ of the third ventricle and distal lateral ventricle regions. We analyzed the numbers of cells in the SVZ, third ventricle lining and electrode region expressing markers of neural precursor cells in normal, PD and PD-DBS brains. In all PD-DBS cases the DBS electrode was chronically implanted and it was more than a year between placement and death. Our results demonstrate a significant increase in the numbers of SVZ neural precursor cells in the ventricles and the area of electrode placement in PD-DBS brains.

## Materials and Methods

All protocols used in these studies were approved by the University of Auckland and the University of Florida Human Participants Ethics committees and informed consent was obtained from all donor families (UF-IRB Project # 130-2008). Tissue from post-mortem human brains used in this study was obtained from one of the following sources: (1) the Neurological Foundation of New Zealand Brain Bank at the Department of Anatomy with Radiology, University of Auckland; (2) DBS tissue samples from the University of Florida Deep Brain Stimulation Brain Tissue Network; or (3) Arizona Parkinson's disease Brain Bank (AZPDB), Sun Valley, Arizona (for details of which cases were from each source, see Table 1). Taken together, the normal cases (n = 10) had a mean age of 76.6±5.9, PD with DBS cases (n = 12) had a mean age of 71.6±6.0, and PD only cases (n = 5) had a mean age of 80.2±4.2. The normal cases were chosen based on the pathology reports (neuropathological examination was carried out on each case by an experienced neuropathologist) that classified the cases as within the normal range for their age with no signs of neuropathological or histological abnormalities. Additionally, the cases were similarly aged, relatively young by human brain study standards and had similar causes of death. The pathology reports for all normal cases revealed that no atrophic changes were observed in the frontal, lateral temporal, hippocampal or cingulate cortices; that there were no plaques or vascular amyloid and no neurofibrillary tangles or excessive glial staining seen with an immunoperoxidase tau stain; that no indications of cortical or nigral Lewy body formation were seen; that there were no infarcts and the small arteries showed no sclerotic changes; that the corpus striatum was unremarkable; and that no significant histological abnormalities were found. The five PD patients without DBS had a clinical history of PD, and the diagnosis of the disease was confirmed by pathological examination. The PD patients subjected to unilateral or bilateral DBS treatment (details of electrode placement are presented in Table 1) had clinically diagnosed PD and pathological examination confirmed the pathology in all cases. Not all brain regions were available for all brains. We chose the cases that had been optimally fixed for each specific study and for which sufficient brain tissue was available to ensure consistency of anatomical region in each case. Because there are regional differences in SVZ proliferation we needed to use blocks that were fixed in the same way and that displayed key anatomical components at previously described. For this reason not all cases were used for counting the progenitor cells in the SVZ. The brains were perfused with PBS containing 1% sodium nitrite followed by 15% formalin fixative in 0.1 M phosphate buffer pH 7.4 for 24 hours. Following perfusion, the brains were dissected, and blocks from the basal ganglia were post-fixed, infiltrated with 30% sucrose and sectioned on a freezing microtome at 50-µm thickness for the cell counting studies in the SVZ. Immunohistochemistry was performed using proliferating cell nuclear antigen (rabbit PCNA – FL-261: Santa Cruz Biotechnology Inc., Santa Cruz, CA, USA; 1∶500) antibody to identify proliferating stem cells in the SVZ. The SVZ overlying the caudate nucleus was divided into equal dorso-ventral thirds, and detailed cell counts of PCNA-positive cells were made at dorsal, middle, and caudal areas of the SVZ as previously described [11]. For the detailed regional cell counting studies, the cases numbered 1–9 on table 1 were used. Two observers blinded to the disease status, area, region and brain from which the image was taken counted the numbers of PCNA-positive cells. Cells were counted from digitised micrographs using ImageJ software. Three rectangular background measurements were taken from areas of the section that did not contain PCNA-positive cells and the background staining from each of these rectangles was averaged. Each cell in the micrograph had a dot placed on it so that no cell was counted twice. Also, a density measurement was taken from the point the dot was located, and recorded for each cell counted. Density measurements, which ranged from 0 to 255 (where 0 = white and 255 = black) were made for each cell; in order for the cell to be counted it had to have a density measurement value of 25 density points higher than the average background measurement. This technique ensured that cells were not included or excluded from the cell counts based on the background density or intensity respectively. Each area sample area was 400 µm in length and cells were counted with the observer blinded to the location and disease status from which the sample was taken.

**Table 1:** Table of cases used in this study showing case number, brain bank the tissue was from, sex, age, stimulator lead placement and side of the brain on which the lead was placed.

	BRAIN TYPE	CASE	ORIGIN	USAGE	AGE	SEX	PM-DELAY	PLACEMENT		DBS to DEATH
1	PD-DBS	DBS25	NZBB	CC	64	M	24	GPi	Bilateral	1 y
2	PD-DBS	DBS195	FDBSTN	CC	67	M	4	STN	Bilateral	5 y
3	PD-DBS	DBS204	FDBSTN	CC	69	M	unknown	STN	Bilateral	6 y
4	PD-DBS	DBS205	FDBSTN	CC	64	F	<24	GPi	Bilateral	6 y R, 1 y L
5	PD-DBS	DBS208	FDBSTN	CC	65	M	∼12	GPi	Left	2 y
6	PD-DBS	DBS210	FDBSTN	CC	70	M	17	STN	Bilateral	0.5 y R, 2 y L
7	PD-DBS	DBS211	FDBSTN	CC	73	M	unknown	VIM	Left	13 y
8	PD-DBS	DBS212	FDBSTN	CC	81	F	<24	STN	Bilateral	5 y R, 6 y L
9	PD-DBS	DBS213	FDBSTN	CC	78	M	<24	STN	Left	3 y
10	PD-DBS	08-70R	AZPDB	CC+PCR/3V+T	75	M	<4	STN	Bilateral	2 y R, 2 y L
11	PD-DBS	08-74L/R	AZPDB	CC+PCR/3V+T	79	F	3.1	STN	Bilateral	3 y R, 3 y L
12	PD-DBS	07-36L/R	AZPDB	CC+PCR/3V+T	75	M	<4	STN	Bilateral	4 y R, 4 y L
13	PD	PD24	NZBB	CC	74	M	7			
14	PD	PD26	NZBB	CC	78	M	7.5			
15	PD	PD37	NZBB	CC	81	M	4			
16	PD	PD42	NZBB	CC	84	M	21			
17	PD	PD52	NZBB	CC	84	M	5			
18	Normal	H151	NZBB	CC	64	F	5			
19	Normal	H156	NZBB	CC	71	M	19			
20	Normal	H158	NZBB	CC	75	M	32			
21	Normal	H393	NZBB	CC	87	F	11			
22	Normal	H6013	NZBB	CC	69	F	11.5			
23	Normal	08-90	AZPDB	CC+PCR/3V	81	M	2.3			
24	Normal	06-62	AZPDB	CC+PCR/3V	82	F	<5			
25	Normal	08-40	AZPDB	CC+PCR/3V	76	M	2.3			
26	Normal	06-66	AZPDB	CC+PCR	78	M	<5			
27	Normal	08-55	AZPDB	CC+PCR	71	M	3			

‘3v’ indicates that tissue was available from the third ventricle and ‘T’ indicates that tissue from these cases was available from the tip of the stimulator lead.

Immunohistochemistry was performed on all brains.

PD-DBS = Parkinson's disease with deep brain electrode placement.

PD = Parkinson's disease.

CC = cell counting.

PCR = polymerase chain reaction.

3V = third ventricle tissue was available and used.

T = tissue from electrode tip was available and used.

NZBB = New Zealand Brain Bank.

FDBSTN = University of Florida Deep Brain Stimulation Tissue Network.

AZPDB = Arizona Parkinson's Disease Bank.

GPi = internal segment of the globus pallidus.

STN = subthalamic nucleus.

VIM = ventrointermediate nucleus of the thalamus.

y = years.

R = right side.

L = left side.

The counts from five consecutive sections per case were accumulated and the mean cell number is presented. Identical anatomical landmarks and procedures were used as previously described by us and only SVZ cells were counted; therefore, ependymal cells did not contribute to the numbers of cells counted [11]. The cell numbers presented here are the average cell number counted in a fixed volume as previously described [11]. The data resulting from the cell counts were analyzed using Prism Version X (Graphpad Software, Palo Alto, CA). A permutation test was performed to compare normal and DBS brains in all regions. The quoted (displayed) values are the means and standard error of the mean for each group. A p-value of less than 0.05 was considered significant. For the tissue purchased from AZPDB, as well as the tissue obtained from the AZPDB, blocks of paraffin-embedded tissue (2 cm×2 cm) from the same anatomical region (subthalamic nucleus at the level of the hypothalamus including a portion of the lining of the third ventricle, and the termination of the electrode lead tip respectively; Table 1) of dissected PD-DBS and control brains were obtained. Eight-micron-thick sections were cut on a microtome (Leica RM 2235) and mounted on coated slides for analysis. For immunohistochemistry, the following primary antibodies were used: Sox2 (mouse, R&D, 1∶500, MAB 2018), MCM2 (rabbit, Cell Signaling, 1: 500, D7G11), and δ-GFAP (rabbit, a generous gift from Dr. Elly Hol, Netherlands Institute for Neuroscience, 1∶1500). Secondary antibodies used were Alexa Fluor 488 donkey anti-mouse IgG (A-21202) and anti-rabbit (A-21206).

Slides were mounted with Vectashield (Vector Labs, H-1200), and analyzed using a Leica DM 2500 fluorescence microscope. Direct comparisons were never made between these 8-µm sections and the 50-µm sections described above. Detailed cell counts of Sox2- and δ-GFAP-positive cells from two sections per case were made at the caudal area of the third ventricle lining. Cell counts of MCM-2-positive cells were made in the region immediately surrounding the electrode lead tip. All statistics were performed using a two-sample permutation test, using the difference in means for each group as the test value.

### qPCR

Four 20-µm sections from each control and PD-DBS brain, from the same anatomical region as described above, were cut on a microtome (Leica RM 2235). The samples were immediately collected in RNAase-free tubes containing xylene and processed for mRNA extraction using the Qiagen FFPE RNA extraction kit (Qiagen, 74404). The concentration of total RNA extracted was measured using a Nanodrop 2000 (ThermoScientific). Only samples with a ratio of absorbance at 260 and 280 nm (A260/280 ratio) of over 1.85 were used for PCR. Following RT-PCR, qPCR using Taqman qPCR primers (Applied Biosystems, Foster City, CA) was performed for the following human genes: *MCM2* and *Sox2* and the housekeeping gene *18S* using Sox2, and MCM2 Taqman gene expression assay kits (Hs01053049_s1 and Hs01091564_m1). Samples were assayed on a real-time qPCR cycler (7900HT, Applied Biosystems) in 96-well optical plates covered with caps. The comparative CT method (ΔΔCT) was employed to determine relative gene level differences between control and DBS tissue, and gene expression was normalized against that for the housekeeping gene *18S*.

## Results

**Figure 1:**
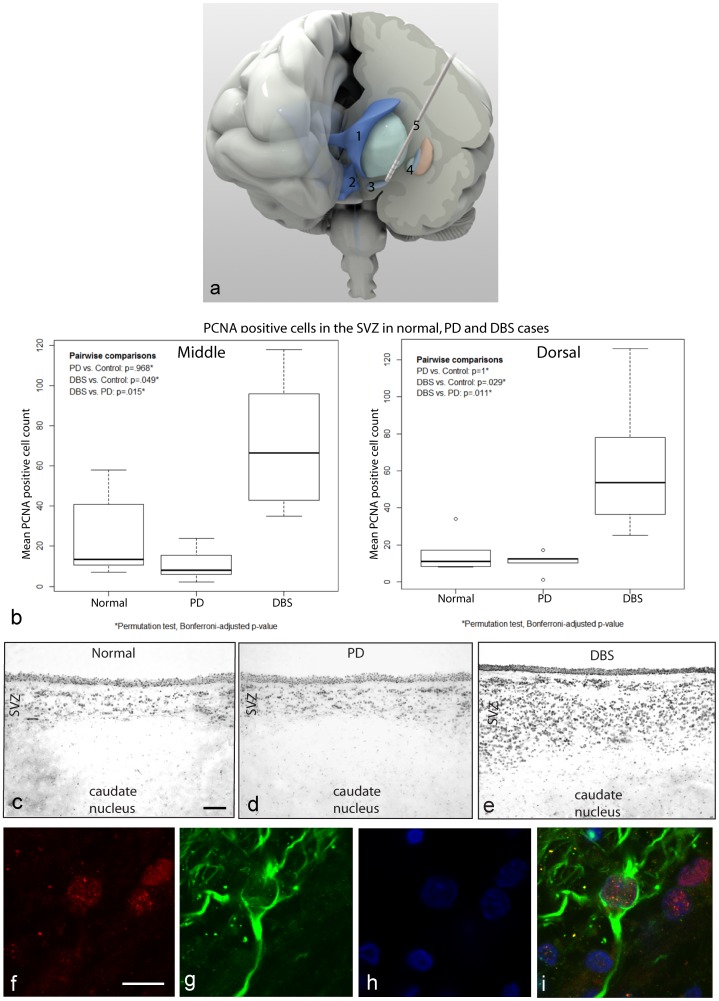
Increased PCNA labeling in the SVZ after deep brain stimulation compared with normal or PD-only brains. **a**. Demonstrates diagrammatically the localization and placement of a deep brain stimulation electrode very close to the subthalamic nucleus just below the globus pallidus. The deep brain stimulation electrode lead sits within close proximity of the lateral and third ventricles. Diagram labeling is as follows: 1 = lateral ventricle, 2 = third ventricle, 3 = subthalamic nucleus, 4 = globus pallidus and 5 = deep brain stimulation electrode. **b**. Graph shows the mean numbers of PCNA-positive cells in the SVZ adjacent to the caudate nucleus of normal, PD and DBS brains. The graph shows a significantly greater number of PCNA-positive cells in brains that received DBS treatment compared with normal brains, but there was no significant difference in the numbers of PCNA-positive cells between normal and PD brains. **c–e**. Overall, the DBS cases show significantly greater mean numbers of PCNA-positive cells compared with normal and PD brains. The figures below the graph illustrate PCNA-positive immunolabelling in the SVZ adjacent to the caudate nucleus of normal, PD and PD-DBS brains. PD-DBS brains had an expanded SVZ compared with normal and untreated PD brains. Scale bar = 100 µm **f–i**. Demonstrates double labeling of PCNA (red) together with δ-GFAP (green) and Hoechst (blue) labeling in the SVZ of a PD-DBS brain. Scale bar = 10 µm.

Detailed analyses of the numbers of PCNA-positive cells in the SVZ adjacent to the caudate nucleus were performed on a total of five normal, five PD and nine PD-DBS brains. In eight of the nine PD-DBS cases, and in one of the five normal brains, both hemispheres were available for study, although no significant difference was seen between left and right (P = 0.279 dorsal and P = 0.410 middle). The dorsal measurements were made from the center of the dorsal 1/3^rd^ of the lateral ventricle and the middle measurements were made from the middle of the middle 1/3^rd^ of the lateral ventricle. For more detail on the anatomical localizations of the regions used for these cell counts see [11]. The average of cell counts from the left and right hemisphere was used for this analysis. The overall mean number of PCNA-positive cells in the SVZ adjacent to the caudate nucleus of PD brains was not significantly different from that in normal brains, but only five PD non-DBS cases were available (Figure 1b). However, there were significantly more PCNA-positive cells in PD-DBS brains (mean ± SEM; dorsal 60.4±10.71; middle 71.14±12.68) compared with normal brains (dorsal 17.5±5.80, P = 0.0252; middle 26.6±9.801; P = 0.0303). There was also a significant difference in the number of PCNA-positive cells between PD and PD-DBS cases (PD dorsal 10.7±5.8, P = 0.011; middle 11.2±8.6, P = 0.015; Figure 1b–e). This corresponded to a 345% (dorsal) and 267% (middle) higher level of proliferation in the PD-DBS SVZ compared with the normal SVZ, respectively. PCNA immunohistochemical labeling of the PD-DBS brains also revealed an expanded SVZ adjacent to the caudate nucleus as compared with normal and untreated PD brains. Many of the PCNA-positive cells in the SVZ were also immunopositive for glial fibrillary acidic protein delta (δ-GFAP) (Figure 1f–i).

**Figure 2:**
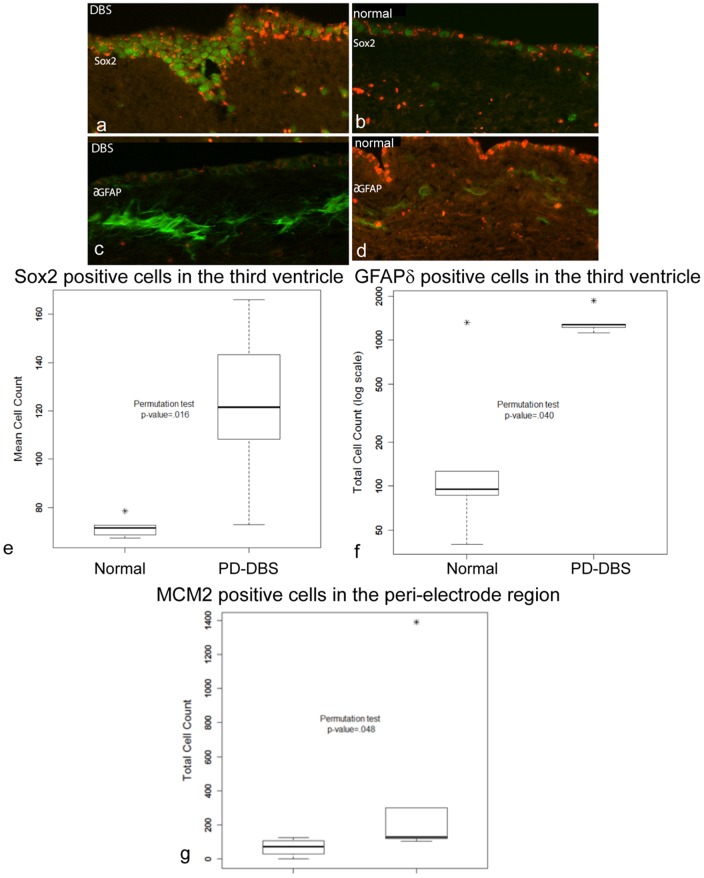
Sox2, MCM2 and δ-GFAP are increased in specific brain regions in PD-DBS brains. The figures illustrate Sox 2-positive labeling in the ependymal layer, adjacent to the hypothalamus, of (**a**) DBS and (**b**) normal brains (in all figures red = autofluorescent blood cells, green = Sox2 or δ-GFAP). The graph (e) shows the mean numbers of Sox2-positive cells in the ependymal layer in normal (negative), and DBS (positive) brain tissue samples. Overall, the DBS-positive cases show a statistically significant difference in the mean numbers of Sox2-positive cells. Scale bar is equivalent for figures a–d = 100 µm. (**c**) The figures illustrate δ-GFAP positive labeling in the ependymal layer, adjacent to the hypothalamus of DBS and (**d**) normal brains. (**e–g**) Boxplots of the cell count data by group. The bold horizontal lines indicate the group medians. The top and bottom of the boxes represent the 75^th^ and 25^th^ percentiles, respectively. An asterisk indicates an outlying data point (a point farther above or below the box than 1.5× the box height). The graph (**e**) shows the mean number of Sox2-positive cells in the ependymal layer in normal and PD-DBS brain tissue samples. The graph (**f**) shows the mean number of δ-GFAP-positive cells in the ependymal layer in normal and PD-DBS brain tissue samples. The graph (**g**) shows the mean number of MCM2-positive cells in the peri-lead region in normal and PD-DBS brain tissue samples.

**Figure 3:**
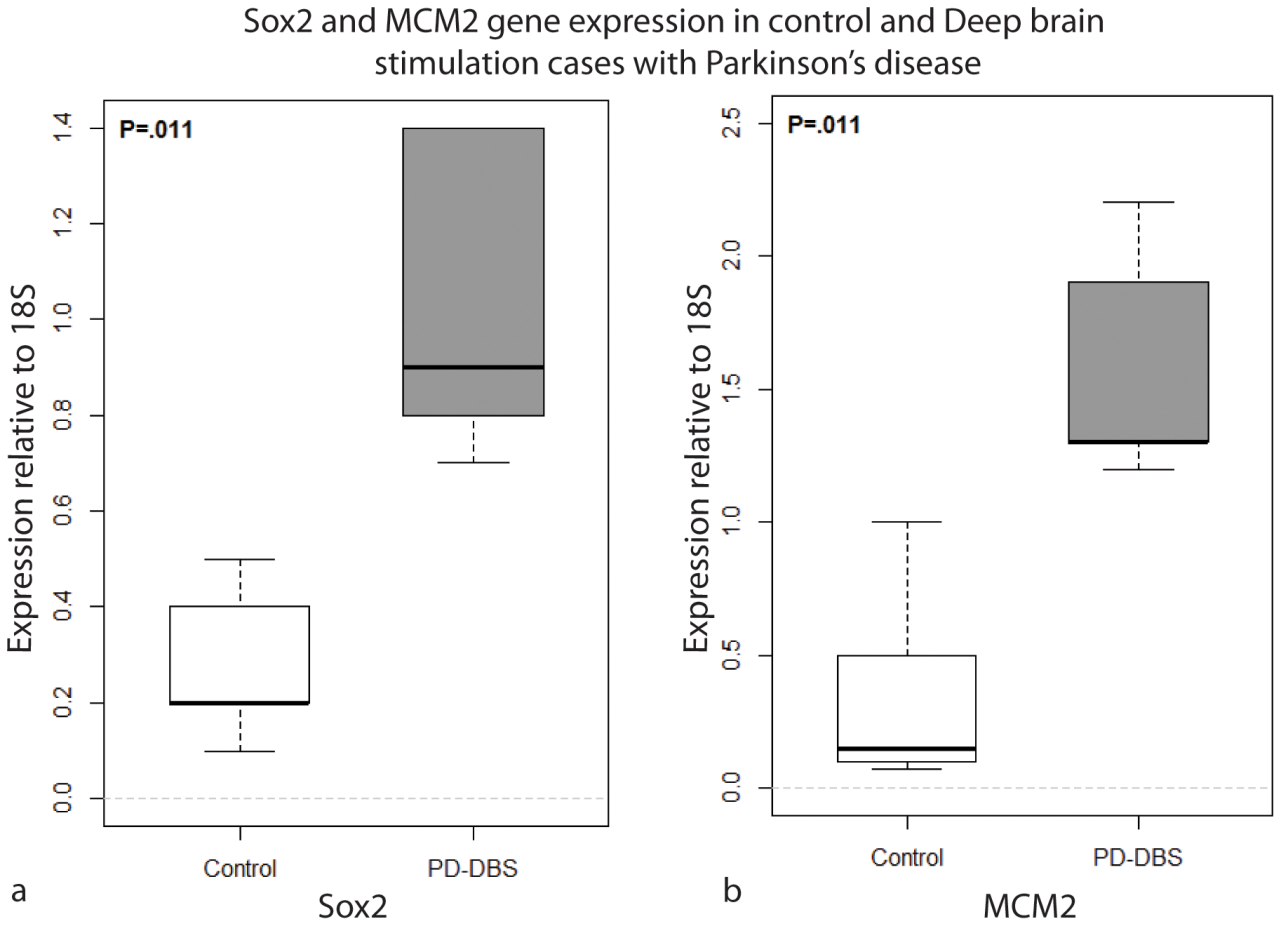
Sox2 and MCM2 gene expression is increased in the lining of the third ventricle and per-lead region in PD-DBS brains. Boxplots of gene expression relative to 18S for Sox2: control and PD-DBS groups. The permutation test p-value shows that the expression level is significantly higher in the PD-DBS group at the 0.05 level. Boxplots of expression relative to 18S for MCM2: control and PD-DBS groups. The permutation test p-value shows that the expression level is significantly higher in the PD-DBS group at the 0.05 level. The relative gene expression level was normalized on the basis of the expression of a reference gene *(18S)* and was also normalized on the basis of the expression of a reference sample (calibrator). Final results are expressed in arbitrary units in gene expression relative to the expression of *18S* gene and calibrator sample. The relative expression was calculated by 2^−ΔΔCT^, where CT = fluorescence threshold value; ΔCT = CT of the target gene – CT of the reference gene *(18S)*; ΔΔCT = ΔCT of the treated sample – ΔCT of the reference sample. A pool of five normal tissue samples served as the reference sample. The increased levels of gene expression of *Sox2* and *MCM2* correlate with the immunolabelling and cell count data.

We then asked whether this higher level of proliferation was exclusive to the highly proliferative SVZ lining the lateral ventricle, or whether such proliferation was a common feature of other areas closely related to the DBS electrode. Owing to the close proximity of the electrode tip to the other neurogenic niche, namely, the third ventricle lining, we chose to study this area and the peri-lead region. Using tissue obtained from the AZPDB, we studied the numbers of proliferating and quiescent precursor cells in the region immediately surrounding the lead tip in PD-DBS human brains (n = 5 hemispheres from 3 cases), and compared them to the numbers in the same regions in normal brains (n = 5). In addition, we quantified the numbers of proliferative precursor cells in the SVZ of the relatively less-studied, but still neurogenic, third ventricle lining (at the level of the hypothalamus) in a subset of brains (case numbers 10–12 and 23–25 in table 1) [22], [23]. This comparison was performed using three markers to label different populations of cells: Sox2 (a transcription factor expressed by precursor cells destined to become glial cells), δ-GFAP (an intermediate filament protein expressed in a subpopulation of astrocytes in the adult human brain ventricles that are putative neural stem cells), and MCM2 (involved in DNA replication at the G1 phase of the cell cycle, and expressed by neural stem cells). We used these three markers to identify cells undergoing cell division, and to identify what proportion were committed to the glial lineage, as evidenced by Sox2 expression, and how many were neural precursors/stem cells based on expression of δ-GFAP. The results revealed statistically significantly greater numbers of Sox2-positive cells (70.87±3.33 normal vs 124.85±5.79 DBS; P = 0.024; Figure 2a, b, g) and δ-GFAP-positive cells (19.63±2.37 normal vs 29.13±1.68 DBS; P = 0.040; Figure 2c, d, f) in PD-DBS brain tissue samples compared with normal brain tissue samples, specifically in the 3rd ventricle. Such a difference was also seen for the numbers of MCM2-immunoreactive cells, specifically in the peri-lead (or equivalent in non-DBS cases) region (Figure 2g, P = 0.048). These differences in the numbers of proliferative cells expressing MCM2 and Sox2 in DBS brains were supported by qPCR results, which revealed higher levels of expression of *Sox2* and *MCM2* genes in the lining of the third ventricle and peri-lead area, respectively, in PD-DBS tissue compared with normal tissue (Figure 3). Together, these findings support the hypothesis that DBS treatment induces proliferation around the DBS lead tip, and stimulates cell division in the SVZ, as shown by the higher levels of expression of genes specifically expressed in neural precursor/stem cells.

## Discussion

This study revealed differences in the numbers of precursor cells in the SVZ overlying the caudate nucleus, the third ventricle and the peri-DBS lead region among post-mortem human PD, PD-DBS, and normal brains. The results indicate the presence of higher numbers of SVZ precursor cells (lining of the lateral ventricle and third ventricle) in DBS brains compared with normal and untreated PD brains. Thus, the intriguing hypothesis arises that the DBS electrodes acted as a stimulant to induce cell proliferation resulting in increased numbers of SVZ precursor cells adjacent to the caudate nucleus, the lining of the third ventricle and the peri-lead regions. The neurodegeneration and transmitter deficiencies associated with PD produce critical disruptions in the activity of a family of basal ganglia circuits known to impact motor and non-motor dysfunction [24]. The neuromodulation of abnormal activity in these circuits through the use of such technologies as DBS can produce important symptomatic benefits and, as we have shown in this study, possibly also alter brain cellular architecture [21]. Our results for the first time provide evidence that apart from the symptomatic relief provided by PD DBS, there is increased proliferation of neural precursor cells in the adult human brain in response to DBS electrode implantation together with electrical stimulation. It is important to keep in mind that there is a very close proximity between the region of stimulation (STN or GPi) and the ventricular system; hence, it would be reasonable to suggest that there is migration of immature NSC-like cells from the SVZ into the peri-DBS region. To date, all of the studied populations of migratory cells have produced glia or interneurons in their target locations; therefore, it is most likely that any neuroblasts that migrate toward the electrode would also become glia or interneurons. However, the possibilities to study this directly are limited because of the temporal loss of migratory and proliferation markers as the neuroblasts become neurons. There are several pathways that project afferently and efferently to the STN and to the striatum, and these pathways could possibly communicate with the SVZ and innervate type C cells, which would potentially drive them to proliferate in the SVZ region [19]. This hypothesis remains speculative. The precise area of tissue stimulation has been estimated and modeled but not specifically measured in all of the tissue that surrounds the activated electrode. However, it is clear from diffusion tensor imaging studies and electrophysiological studies that the extent of spread of stimulation from the activated electrodes is dependent on the number of heavily myelinated fibers in the peri-electrode region. Under normal circumstances tissue activation is estimated to occur up to 4–5 mm away from the electrode tip [25], [26]. The distance from the medial aspect of the STN to the third ventricle is 4–7 mm [27]. This close proximity, therefore, makes it possible that the higher levels of proliferation observed are a direct effect of DBS. We cannot rule out the possibility that electrode insertion plays a role in the observed findings given that any puncture injury to the brain elicits a short-term (up to about 14 days) proliferative response. However, we have studied the brain of PD-DBS patients that died some considerable time (ranging from 0.5 to 6 years) after electrode placement, and thus, it is unlikely that the increase in progenitor proliferation is a result of the initial surgical intervention [28], [29]. We also cannot rule out the possibility that chronic electrode placement leads to the proliferative response seen. The mechanism of action of the high-frequency stimulation employed in DBS has not yet been fully unraveled, although DBS has been used clinically to treat PD for over two decades. Most experts believe that DBS acts in both inhibitory and excitatory fashions, and that, in addition to physiological changes, there is propagation of a calcium wave that leads to neurotransmitter release and changes in blood flow. Based on these possible effects of DBS, there are four main hypotheses that have been proposed to explain the mechanism of action of DBS: synaptic inhibition [4], synaptic depression [30], depolarization blockade [31], and modulation of network activity induced by stimulation [32]. There is one report indicating that STN DBS is neuroprotective, or has disease- modifying effects, although this is a topic that is highly debated in the field [3]. If disease modification does occur as a result of DBS, we know that it must be minimal, as PD is known to progress despite DBS [33], [34]. If disease modification did occur on a small scale, our hypothesis would be that it is achieved, at least in part, due to the proliferative effect of the electrical stimulation in the NSC/precursor cell compartment in the adult brain. Although our observations of proliferative NSCs are insufficient to argue for the prevention of the progression of PD, they do provide a possible strategy to stimulate a system that can endogenously repair the damaged brain. It is our hope that, through these findings, endogenous neural stem cell proliferation and potential mobilization from germinal niches can in the future be harnessed for the treatment of neurodegenerative diseases such as PD, Alzheimer's disease, Huntington's disease, amyotrophic lateral sclerosis, dystonia, and stroke.
